# Assessment of the knowledge of teachers about asthma and the availability of facilities for asthma care in public secondary schools in Lagos, Nigeria

**DOI:** 10.7196/AJTCCM.2018.v24i2.192

**Published:** 2018-06-21

**Authors:** O O Adeyeye, Y A Kuyinu, O B Ozoh

**Affiliations:** 1 Department of Medicine, Lagos State University College of Medicine, Lagos, Nigeria; 2 Department of Community Medicine and Primary Health Care, Lagos State University College of Medicine, Lagos, Nigeria; 3 Department of Medicine, College of Medicine, University of Lagos, Lagos, Nigeria

**Keywords:** asthma: school care, knowledge, teachers, facilities

## Abstract

**Background:**

Asthma is a common chronic illness affecting young people. Asthma management at schools may be influenced by teachers’ knowledge of the condition and the availability of treatment facilities.

**Objectives:**

To assess the knowledge of secondary school teachers in Lagos, Nigeria, regarding asthma and to evaluate management options available at schools.

**Methods:**

A descriptive cross-sectional study was conducted. Schools were selected by proportional sampling of the educational districts, followed by stratified single-stage cluster sampling. All consenting teachers in the 54 selected schools were recruited. A self-administered questionnaire was used for data collection. A composite score was calculated, with 32 as the maximum possible. Knowledge was regarded as poor if scores were <16, fair for scores between 16 and 21, and good if scores were ≥22.

**Results:**

Results show that 475 (48.1%) of the respondents had poor knowledge, 414 (41.9%) had fair knowledge, and only 99 (10%) had good knowledge. Better knowledge about asthma was associated with personal experience (χ² =16.466; p=0.001) or history of a family member with the condition (χ² =6.412; p=0.04). Of the 54 schools surveyed, only 9 (16%) had a school clinic, while a school nurse was available at only 4 (7.41%) of the schools. None of the schools had access to a nebuliser in case of an asthma emergency.

**Conclusion:**

Teachers in secondary schools in Lagos have unsatisfactory knowledge about asthma and are not equipped to support affected students during an asthma episode.

## Background

Asthma is a common chronic condition among children and young adults. It is estimated that 235 million people worldwide have asthma and that the condition was responsible for about 383 000 deaths in 2015.^[1]^ According to the International Study of Asthma and Allergies in Childhood (ISAAC),^[2]^ the prevalence of asthma among 13 - 14-year-olds in Nigeria was 10.7% in 1998 and has increased to 13% in 2002.^[3]^

The management of non-communicable diseases such as asthma is not given priority by policy makers in Nigeria. This may be due, in part, to the high burden of communicable diseases and limited healthcare resources. In an earlier report, skin problems, malnutrition and respiratory tract illnesses were noted as the leading health problems among pupils in Lagos.^[4]^ Although asthma was not specifically mentioned, it is likely that asthma contributed to the burden of respiratory illnesses.

Asthma is one of the leading causes of absenteeism from school and work and often limits participation in physical and social activities, including exercise. In the USA, asthma is estimated to cause approximately 14 million absent school days each year.^[5]^ There are no comparable data about absent school days due to asthma in Nigeria.

Students with asthma can develop acute emergencies during school hours if exposed to triggers or when participating in exercise during sporting activities. The competence of teachers with regard to first aid, the availability of treatment facilities and the presence of competent healthcare personnel may influence the outcome of such episodes. Delayed or inappropriate response to asthma emergencies has been reported to lead to asthma deaths in schools.^[6]^

In many parts of the world, teachers’ knowledge regarding asthma has been reported to be only modest (about 40 - 70%) and lower still in disadvantaged communities.^[7–10]^ To our knowledge, Nigerian teachers’ knowledge regarding asthma has not been reported nor have the resources for asthma care in schools been evaluated.

Our study therefore investigated the level of knowledge about asthma among teachers in secondary schools in Lagos, Nigeria. It also described the factors associated with the level of knowledge among the teachers and evaluated the availability of facilities and personnel in schools to support asthmatic students during emergencies. As public schools serve a wide range of students, including those with limited access to basic healthcare, the findings from this study may help to make schools safer for students with asthma.

## Methods

This was a descriptive cross-sectional study among teachers of selected schools operating under the Lagos State Government Ministry of Education. The study was conducted between August and December 2016 in 54 public secondary schools in Lagos, which represent 20% of all the urban secondary schools in the city.

**Fig. 1:**
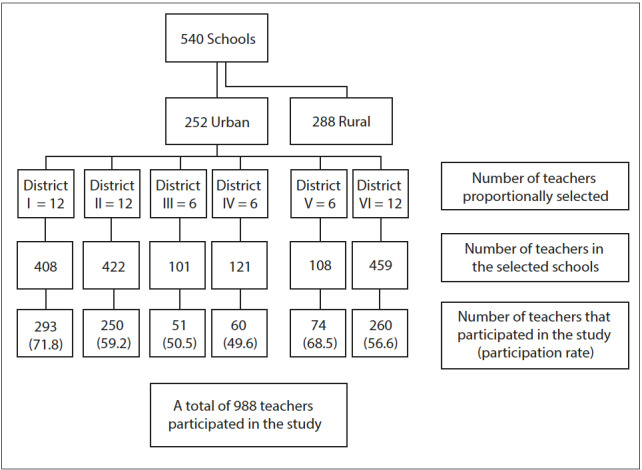
Flow chart for the selection of teachers from educational districts.

The six educational districts in Lagos were used as sampling units. We obtained a list of public secondary schools in these districts and stratified them into urban and rural schools. We systematically selected the schools to be sampled from the 252 urban schools (Fig. 1).

The schools were numbered per district and the second one in each district list was used as starting point. Every fifth school in a district was subsequently selected until the desired number of schools was obtained. As the districts do not all have an equal number of schools, the number of schools selected in a district was proportional to the total number of urban schools in that district. All the teachers in a selected school were eligible to participate in the study and all those who consented were included.

The sample size was calculated according to the formula for determining proportions in a cluster survey of at least 20 000 teachers.^[11]^ The assumption was that 50% of the teachers would have adequate knowledge. The minimum sample size was calculated as 660 teachers. We selected 30 teachers from each of at least 22 schools. A design effect size of 1.5 and a significance level of p<0.05 were used.

A 47-item questionnaire, validated through use in previous studies,^[12–14]^ was used to collect information on sociodemographic characteristics and personal history of asthma. Questions explored the teachers’ knowledge of asthma triggers, symptoms, severity, treatment options and associated conditions. The questionnaire also assessed common myths about asthma and teachers’ willingness to participate in an educational intervention programme.

The proportion of correct responses was used as outcome. A score of 1 was assigned for a correct response to a question, whereas incorrect responses or ‘don’t know’ answers were assigned a score of 0. A composite score was then calculated, with the maximum attainable score being 32. Knowledge level was rated as poor if the composite score was <16, fair for scores between 16 and 21, and good for scores ≥22.

A second questionnaire, completed by school principals, was used to assess the availability of treatment facilities at the school, such as a school clinic, access to a nebuliser, spare inhaler or spacer device, or the availability of a school nurse.

### Statistical analysis

Data were analysed using SPSS version 24 (IBM Corp., USA). Means were calculated for all quantitative data and expressed as mean (standard deviation). The χ² test was used to compare categorical variables, whereas quantitative data were compared using Student’s t test. A significance level of p≤0.05 was used.

### Ethics

Ethical approval for the study was obtained from the Health Research Ethics Committee of the Lagos State University Teaching Hospital (ref. no. LREC/10/06/1022), the authorities at the Lagos State Ministry of Education (ref. no. LED/BES/S.191/T2/248) and the district tutors general. Informed consent was obtained from each school principal and the individual participating teachers.

## Results

Of the 1 619 teachers in the districts, 988 completed the questionnaire acceptably. This translates to a participation rate of 61%. Sociodemographic characteristics are shown in Table 1. The sample comprised 355 male teachers (35.9%) and 633 female teachers (64.1%). The mean age of the teachers was 44.6 (8.7) years (range: 19 - 69 years). Most of the teachers had at least a Bachelor’s degree and an average of 17.2 (8.8) years’ post qualification teaching experience. The prevalence of previously diagnosed asthma among the teachers was 5%. A third of the respondents had a first-degree relative with asthma.

Responses from the questionnaire showed that 735 (74.4%) of the teachers had heard about asthma before and 350 (35.5%) were aware of a student with asthma in their school.

**Table 1:** Sociodemographic characteristics of respondents (N=988)

**Variables**	**Frequency, *n*(%)**
**Age (years)**	
<20	4 (0.4)
21 - 30	73 (7.4)
31 - 40	226 (22.9)
41 - 50	424 (42.9)
51 - 60	260 (26.3)
61+	1 (0.1)
Mean (SD) age: 44.6 (8.7)	
**Gender**	
Male	355 (35.9)
Female	633 (64.1)
**Marital status**	
Married	845 (85.5)
Widowed	32 (3.2)
Divorced	2 (0.2)
Separated	5 (0.5)
Not specified	9 (0.9)
**Subject taught**	
Art	307 (31.1)
Social science	286 (28.9)
Pure/applied science	13 (1.3)
**Post-qualification experience (years)**	
≤5	102 (10.3)
6 - 10	210 (21.3)
11 - 15	111 (11.2)
16 - 20	170 (17.2)
>20	395 (40)
Mean (SD) post qualification experience: 17.2 (8.8)	
**Personal history of asthma**	
Yes	49 (5.0)
No	932 (94.3)
**Family history of asthma**	
Yes	363 (36.7)
No	624 (63.2)

SD = standard deviation

Teachers’ responses to questions about asthma symptoms, severity and comorbidities are shown in Table 2. About 40% of the teachers agreed that asthma is a common respiratory disorder in children and about one-third (30.9%) knew that asthma can be associated with an allergy. Two-thirds (66.2%) of the respondents thought, incorrectly, that asthma is curable and three-quarters (75.2%) correctly indicated that asthma symptoms can be controlled with the use of proper medication.

When asked about the symptoms of asthma, 644 (65.2%) respondents recognised persistent cough as a common symptom and an almost similar proportion (68.7%) knew that speech difficulty may occur during an asthma attack. About 20% of the teachers recognised a recurrent runny nose and an itchy skin rash as possible comorbidities in asthma.

**Table 2:** Responses of secondary school teachers regarding asthma symptoms, severity and comorbidities (N=988)

	**Yes,**	**No,**	**Do not know,**
**Survey question**	***n* (%)**	***n* (%)**	***n* (%)**
Asthma is a common respiratory disorder in children.	391 (39.6)	322 (32.6)	275 (27)
Allergies are associated with asthma.	305 (30.9)	259 (26.2)	424 (42.9)
Children with asthma have a low intelligent quotient (IQ).	213 (21.6)	375 (38.1)	400 (40.5)
Asthma is curable.	654 (66.2)	84 (8.5)	250 (25.3)
Asthma can be controlled with proper use of medication.	753 (75.2)	54 (5.5)	191 (19.3)
Persistent cough may be a presentation of asthma.	644 (65.2)	115 (11.6)	229 (23.2)
Asthma may cause speech difficulty during an attack.	679 (68.7)	75 (7.6)	234 (23.7)
Rapid breathing in a child may result from asthma.	528 (53.4)	108 (10.9)	352 (35.6)
Noisy breathing may occur in a child with asthma.	559 (56.6)	119 (12.0)	310 (31.4)
Chest discomfort may be a complaint in asthma.	505 (57.2)	153 (15.5)	329 (33.3)
Asthma symptoms may develop in an otherwise healthy child.	411 (41.6)	163 (16.5)	414 (41.9)
Agitation and restlessness may signify worsening of a symptom.	469 (47.5)	88 (8.9)	431 (43.6)
Drowsiness and confusion may signify a severe problem in asthma.	526 (53.2)	93 (9.4)	369 (37.3)
Students with asthma may have itchy eyes.	767 (77.6)	59 (6.0)	160 (16.2)
Students with asthma could have a recurrent runny nose.	214 (21.8)	181 (18.3)	593 (60)
Students with asthma could have an itchy skin rash.	215 (21.8)	115 (11.6)	658 (66.6)
Students with asthma could exhibit other allergies, including food allergies.	287 (29)	330 (34)	365 (36.9)

Teachers’ responses to questions regarding triggers and treatment are shown in Table 3. A large proportion of the teachers were aware of potential triggers of asthma episodes. Smoke was indicated as a trigger by 841 (85.1%) of the respondents, while 685 (69.3%) and 632 (64%) of respondents indicated that chalk dust and cold, respectively, can trigger an asthma episode. The majority of the respondents knew about the reliever medication Ventolin. About 90% of the teachers recognised the need to train teachers about asthma, while 909 (92%) were willing to participate in such training.

**Table 3:** Responses of teachers regarding asthma triggers and treatment (N=988)

	**Yes,**	**No,**	**Do not know,**
**Survey question**	***n* (%)**	***n* (%)**	***n* (%)**
Exposure to smoke leads to symptoms of asthma.	841 (85.1)	55 (5.1)	92(9.3)
Exposure to chalk dust leads to symptoms of asthma.	685 (69.3)	127 (12.9)	171 (17.8)
Exercise leads to symptoms of asthma.	490 (49.6)	160 (16.2)	338 (34.2)
Exposure to cold leads to symptoms of asthma.	685 (69.3)	72 (7.3)	231 (23.4)
Exposure to dust leads to symptoms of asthma.	632 (64)	51 (5.2)	305 (30.8)
Some over-the-counter medication for pain may lead to an asthma attack.	288 (29.1)	113 (11.4)	587 (59.4)
Does the use of antibiotics relieve an asthma attack?	439 (44.4)	92 (9.3)	447 (44.3)
Does a salbutamol/Ventolin inhaler relieve an asthma attack?	786 (79.6)	21 (2.1)	181 (18.3)
Asthmic children require preventive treatment.	752 (76.1)	149 (15.1)	86 (8.8)
Blue inhalers are used in an emergency.	600 (60.9)	27 (2.7)	361 (36.5)
A severe asthma attack should be managed in hospital.	614 (62.1)	93 (9.4)	281 (284)
Asthma can be managed with native medications.	349 (35.3)	215 (21.8)	424 (42.9)
Children with asthma should not engage in sports.	536 (54.3)	148 (15)	304 (30.8)
Asthmatic students should use their inhalers before exercise.	547 (55.4)	117 (11.8)	323 (32.8)
Keeping the school free of pets is beneficial to asthmatic students.	767 (77.6)	59 (6.0)	160 (16.2)
Keeping the classroom dust free is beneficial to an asthmatic child.	671 (67.9)	61 (6.2)	256 (25.9)
Had previous training on asthma care.	377 (38.2)	611(61.8)	

A mean knowledge score of 15.31 (5.74) was achieved by the sample. Of the total number of respondents, 475 (48.1%) achieved a score below 15, indicating poor knowledge, while 414 (41.9%) achieved a score between 16 and 22, indicating fair knowledge. Only 99 (10%) of the teachers showed good knowledge (Table 4).

Personal history of asthma was associated with better knowledge (χ² =16.466, p=0.001), as was a family history of asthma (χ² =6.412; p=0.04) and having a student with asthma (Table 4). None of the other factors listed in Table 4 were found to have a significant association with knowledge level (p>0.05).

Only 9 (16%) of the schools visited had a clinic on the premises and a school nurse was available at only 4 (7.4%) of the schools. None of the schools had access to spare reliever medication, spacers or a nebuliser.

**Table 4:** Association between sociodemographic characteristics of secondary school teachers in Lagos, Nigeria, and their knowledge level about asthma (N=988)

	**Knowledge level, *n* (%)**				
**Sociodemographic characteristics**	**Poor (n=475)**	**Fair (n=414)**	**Good (n=99)**	**χ²/t**	***p*-value**
**Age (years)**				16.428	0.172
<20	1 (0.2)	3 (0.6)	0 (0)		
21 - 30	42 (8.8)	23 (5.6)	8 (8.1)		
31 - 40	119 (25.1)	86 (20.8)	21 (21.2)		
41 - 50	209 (44.0)	172 (41.5)	43 (43.4)		
51 - 60	104 (21.9)	129 (31.2)	27 (27.3)		
61+	0 (0)	1 (0.2)	0 (0)		
**Gender**				3.128	0.20
Male	184 (51.8)	138 (38.9)	33 (9.3)		
Female	291 (46)	276 (43.6)	66 (10.4)		
**Level of education**				13.292	0.208
National Certificate of Education	32 (6.7)	27 (6.5)	7 (7.1)		
Bachelor’s degree	344 (72.4)	282 (68.1)	58 (58.6)		
Diploma in Education	24 (5.1)	24 (5.8)	6 (6.1)		
Master’s degree	67 (14.1)	70 (16.9)	22 (22.2)		
Doctorate	1 (0.2)	3 (0.3)	1 (0.1)		
Not specified	7 (1.5)	8 (1.9)	5 (5.1)		
**Marital status**				5.490	0.856
Single	52 (10.9)	38 (9.2)	5 (5.1)		
Married	402 (84.6)	355 (85.7)	88 (88.9)		
Widowed	15 (3.2)	13 (3.1)	4 (4.0)		
Divorced	1 (0.2)	1 (0.2)	0 (0)		
Separated	1 (0.2)	3 (0.7)	1 (1.0)		
Not specified	4 (0.8)	4 (1.0)	1 (1.0)		
**Post-qualification experience (years)**				17.760	0.023
≤5	56 (11.8)	36 (8.7)	10 (10.2)		
6 - 10	109 (22.9)	85 (20.5)	16 (16.3)		
11 - 15	58 (12.2)	45 (10.9)	8 (8.2)		
16 - 20	92 (19.4)	59 (14.3)	19 (19.4)		
>20	160 (33.7)	189 (45.7)	45 (45.9)		
**Personal history of asthma**				16.466	0.001
Yes	13 (2.8)	24 (5.8)	12 (12.2)		
No	459 (97.2)	387 (94.2)	86 (87.8)		
**Relative with asthma**				6.412	0.04
Yes	188 (40.6)	148 (36.7)	27 (27.3)		
No	275 (59.4)	255 (63.3)	72 (72.7)		
**Student(s) with asthma**				8.778	0.012
Yes	146 (32.2)	169 (41.9)	35 (36.8)		
No	308 (67.8)	234 (58.1)	60 (63.2)		

## Discussion

Our results show that knowledge about asthma was poor among secondary school teachers in Lagos, Nigeria. This may have serious consequences, as an unrecognised asthma attack may prove potentially fatal.

It is surprising that although more than three-quarters of the teachers have heard about asthma before, most were unaware of students being affected by asthma. This may lead to their failing to recognise symptoms and inadequate responses to the need of the students.

The mean score in this survey was 15.31 (5.74) out of a possible 32. This is similar to the findings from a Bahraini study, in which teachers scored an average of 5.16 (2.18) out of 10.^[15]^ The finding is also similar to that of a Spanish study, in which the mean score from a sample of 7 000 teachers recruited from 9 cities, was 16.0 (4.8) out of 31.^[16]^ Only 10% of the teachers in our study showed good knowledge about asthma, which is unlike the findings from a Pakistani study in which more than half the teachers surveyed had good knowledge.^[17]^ It should be noted, however, that the sample size in that study was only 330 teachers, which may render this comparison contentious.

The low knowledge score in our study may be a reflection of the general lack of awareness about asthma in Nigeria and possibly be related to the chronic nature of asthma not being widely recognised.

The prevalence of physician-diagnosed asthma among the teachers in our study was 5%, which compares well with the 5.8% found in a Spanish study.^[16]^

As in some other studies, our results also showed that a personal history of asthma or having a relative with asthma was associated with better knowledge about the condition.^[15–18]^ This was an expected finding, as exposure to health education in the course of their illness or as carers to affected family members may have translated to better knowledge and increased awareness among these teachers.

In our study, neither gender nor teachers’ level of qualification affected the scores. This is different from the Pakistani study,^[17]^ which found female teachers and those with a Bachelor’s degree or higher to have better knowledge about asthma.

There was no evidence of formal training opportunities to equip teachers with knowledge to deal with students’ health concerns. However, about a third of the teachers indicated having had exposure to some informal education initiatives, mostly obtained to guide personal care or care of a first-degree relative with asthma.

Our study revealed some misconceptions and knowledge gaps about the symptoms, triggers and management of asthma. Many teachers indicated that they thought asthma is a curable condition. This assumption could influence the type of advice teachers give to parents and so may further propagate the misconception, as teachers are respected opinion leaders in their communities. As they provide advice to parents, teachers with poor knowledge about asthma may provide wrong advice about seeking help, which may negatively affect the health of a student. It is therefore important that knowledge gaps with regard to treatment options be addressed through effective educational intervention.

Some teachers indicated that they consider poor academic performance of students with asthma to be a result of asthmatic students inherently having a low intelligent quotient; this is not supported by any evidence. The poor scholastic performance may be due to frequent absenteeism. Good asthma control can be achieved if appropriate support is available at school to deal with episodes and create an asthma-friendly school environment. This will reduce absenteeism and promote learning.

Questions about potential triggers of asthma revealed variable knowledge. Many respondents recognised exposure to smoke as a trigger, but not that some over-the-counter analgesics may also trigger an attack. This may lead to an unnecessary asthma attack if a student were to receive an analgesic for pain relief at school. Several teachers also did not show satisfactory knowledge about exercise-induced asthma. This is similar to the findings of Aqeel *et al*.^[17]^ but different from those of Hussey *et al*., ^[18]^ who reported about 80% of their teachers knowing about exercise-induced asthma. Asthmatic students may subsequently be excluded from sporting activities, which may, in turn, promote an inactive lifestyle and unsatisfactory management of the condition.

Only half of the respondents in our study could recognise symptoms of an asthma attack. This is different from the findings of Aqeel *et al*., ^[17]^ who reported that about 80% of their respondents showed good knowledge about the symptoms of an asthma attack. The high level of knowledge seen in that study was attributed to teachers having access to diverse information sources about asthma. We could not find evidence that teachers had access to additional information about asthma other than what was provided in the case of a personal history of asthma or if an asthmatic relative had to be cared for.

We also found poor knowledge among respondents about other conditions that may coexist with asthma. Training teachers to recognise when medical assessment would be needed may prove valuable, particularly in resource-poor settings where only limited healthcare professionals are available.

Although about 80% of the respondents in our study knew that an inhaler could be used to relieve the symptoms of asthma, we did not explore whether the teachers had experience in using an inhaler.

## Conclusion

Owing to the poor knowledge of teachers about asthma and the lack of appropriate facilities, schools in Lagos will unlikely be able to offer satisfactory support to students with asthma. It is recommended that teachers be skilled appropriately through training initiatives to address the situation and that school clinics be equipped with basic emergency medication to deal with an asthma episode.

## References

[1] World Health Organization Asthma. http://www.who.int/news-room/fact-sheets/detail/asthma.

[2] International Study of Asthma and Allergies in Childhood (ISAAC) Steering Committee. (1998). Worldwide variation in prevalence of symptoms of asthma, allergic rhinoconjunctivitis, and atopic eczema.. Lancet.

[3] Mallol J, Crane J, Von Mutiu E, Odhiambo J, Keil U, Stewart A (2013). The International Study of Asthma and Allergies in Childhood (ISAAC) Phase Three: A global synthesis.. Allergol Immunopathol.

[4] Lagos State Ministry of Education 2011 and 2012 State of Education Reports in Lagos State. http://www.lasgmoed.com/wp-content/uploads/2014/02/2011-12-SoER-Final6-1-corrected-final_PF-2014.pdf.

[5] Five health-related causes of chronic absenteeism. Healthy Schools Campaign [Internet].

[6] Greiling AK, Boss LP, Wheeler LS (2005). A preliminary investigation of asthma mortality in schools.. J Sch Health.

[7] Unikel LH, Evans D, Bornstein L, Surrence K, Mellins RB (2010). Asthma knowledge and asthma management behavior in urban elementary school teachers.. J Asthma.

[8] Getch YQ, Neuharth-Pritchett S (2009). Teacher characteristics and knowledge of asthma.. Public Health Nurs.

[9] Szczepanski R, Brockmann G, Friede G (2001). [Dealing with asthma for teachers – needs and possibilities].. Pneumologie.

[10] Henry RL, Gibson PG, Vimpani GV, Francis JL, Hazell J (2004). Randomized controlled trial of a teacher-led asthma education program.. Pediatr Pulmonol.

[11] Dean AG, Sullivan KM, Soe MM OpenEpi: Open Source Epidemiologic Statistics for Public Health.. http://www.openepi.com/.

[12] Pitstick C (2014). Asthma knowledge among primary and secondary school teachers in rural northern Costa Rica.. UNED Research J.

[13] Govender D, Gray A (2012). Knowledge of primary school teachers about asthma: A cross-sectional survey in the Umdoni sub-district, KwaZulu-Natal.. S Afr Fam Pract.

[14] Brookes J, Jones K (1992). Schoolteachers’ perceptions and knowledge of asthma in primary schoolchildren.. Br J Gen Pract.

[15] Alnasir FAL (2004). Bahraini school teachers’ knowledge of asthma.. Middle East J Fam Med.

[16] Varela AL, Esteban SR, Díaz SP (2016). Knowledge of asthma in school teachers in nine Spanish cities.. Pediatr Pulmonol.

[17] Aqeel T, Akbar N, Dhingra S, Haq N-U (2015). Assessment of knowledge and awareness regarding asthma among school teachers in urban area of Quetta, Pakistan.. J Pharm Pract Comm Med.

[18] Hussey J, Cahill A, Henry D, King AM, Gormley J (1999). National school teachers’ knowledge of asthma and its management.. Ir J Med Sci.

